# Total neoadjuvant therapy in oesophageal and gastro-oesophageal junctional adenocarcinoma

**DOI:** 10.1038/s41416-023-02458-w

**Published:** 2023-10-28

**Authors:** Hollie A. Clements, Tim J. Underwood, Russell D. Petty

**Affiliations:** 1 Division of Molecular and Clinical Medicine, Ninewells Hospital and Medical School, University of Dundee, Dundee, UK; 2 School of Cancer Sciences, University of Southampton and University Hospital Southampton NHS Foundation Trust, Southampton, UK; 3 Tayside Cancer Centre, Ninewells Hospital and Medical School, NHS Tayside, Dundee, UK

**Keywords:** Oesophageal cancer, Oesophageal cancer

## Abstract

Adenocarcinoma of the oesophagus and gastro-oesophageal junction represent a large burden of cancer death in the Western World with an increasing incidence. In the past two decades, the overall survival of patients on a potentially curative treatment pathway has more than doubled due to the addition of perioperative oncological therapies to surgery. However, patients often fail to respond to oncological treatment or struggle to complete their treatment after surgery. In this review, we discuss the current evidence for total neoadjuvant therapy and options for assessment of treatment response.

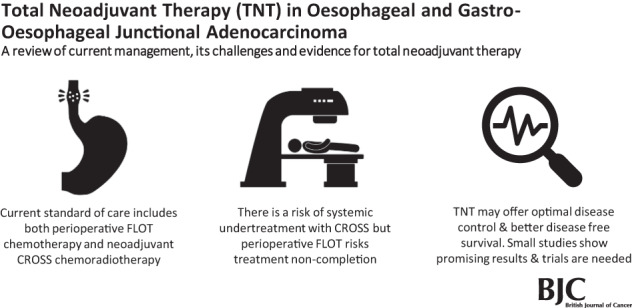

## Background

Oesophageal cancer is the 14th most common cancer in the United Kingdom, with adenocarcinoma being the most common histological subtype, and it is the 7th most common cause of cancer death [1]. The overall benefit of preoperative or perioperative oncological therapy (chemotherapy or chemoradiotherapy) for oesophageal and junctional adenocarcinoma is widely recognised, most notably demonstrated by landmarks trials including OEO2, MAGIC, FLOT4 and CROSS with overall 5-year survival reaching 47% in CROSS, a large improvement from 17–23% for surgery alone [2–5]. In this review, we discuss the effect of neoadjuvant chemotherapy (nCT) and chemoradiotherapy (nCRT) on surgical resection margin, lymph node downstaging, and primary tumour pathological response and how this impacts survival. We also review the challenges of delivering perioperative therapy and discuss total neoadjuvant therapy as a potential novel treatment regimen for patients with resectable oesophageal cancer.

## Total neoadjuvant therapy—is it possible in oesophageal cancer?

Total Neoadjuvant Therapy (TNT), where all oncological treatment is delivered before surgery, is beneficial in other cancer types. This experience should be exploited with regard to potential issues which could arise using the TNT approach for oesophageal cancer, such as increased toxicity and poorer tolerability of neoadjuvant therapy given the higher chemotherapy dose and subsequent failure of progression to surgery.

For example, TNT has gained prominence in the management of locally advanced rectal cancer and has now been incorporated into national rectal cancer guidelines [6]. A summary of key trials of TNT in rectal cancer is shown in Table 1. The RAPIDO trial included high-risk patients with T4 or N2 clinical staging or other high-risk factors, and patients in the TNT group received 6 cycles of CAPOX or FOLFOX4 chemotherapy following a short course of radiotherapy [7]. In the UNICANCER-PRODIGE 23 trial, patients with T3-T4 and N0 clinical disease were included, arguably less advanced disease than in RAPIDO [8]. This trial used the more aggressive FOLFIRINOX chemotherapy before nCRT and showed a better 3-year disease-free survival in the TNT group and the trial concluded that there was a lower toxicity rate in the TNT group despite using FOLFIRINOX. These trials did not show any improvement in overall survival with TNT, but two meta-analyses have supported the improved oncological outcomes using TNT and consensus has shifted in favour of TNT in locally advanced rectal cancer [9, 10]. As well as these trials, there are various other smaller studies of TNT in rectal cancer, which consistently show better tolerability, less toxicity, and higher rates of planned treatment completion when the TNT approach is used [11–13]. Furthermore, excellent results have been reported in Phase 2 trials of TNT for borderline resectable pancreatic adenocarcinoma, with 2-year progression-free and overall survival of 43% and 56%, respectively and a median PFS of 48.6 months in those undergoing resection with 2-year OS of 72% [14]. There is evidence to suggest that TNT enables the delivery of intended systemic therapy with a greater chance of pCR and without compromising on surgical resection in pancreatic adenocarcinoma [15]. A recent systematic review and meta-analysis suggest superior oncological and pathological outcomes with TNT compared to standard neoadjuvant therapy [16]. Phase III trials of TNT in pancreatic adenocarcinoma are currently ongoing [17].

**Table 1:** A summary of key trials of total neoadjuvant therapy in rectal cancer.

Trial name	Study type	Patients (n)	Inclusion	Treatment arm A	Treatment arm B	Toxicity, resection & surgical complications	Pathological/survival outcomes
POLISH II (2016) [94, 95]	Randomised, Phase III	515	cT3 or cT4 primary or recurrent rectal cancer	**TNT:** SCRT (5x5Gy) + 3 cycles FOLFOX4 then TME	CRT (50.4Gy + 5-FU, leucovorin + oxaliplatin) then TME	Toxicity: 75% vs 83% (*P* = 0.006) R0: 77% vs 71% (NS) Postoperative complications: 29% vs 25% (NS)	pCR 16% vs 12% (NS) 3Y OS: 73% vs 65% (*P* = 0.046) 3Y DFS: 53% vs 52% (NS) 8Y OS: 49% (both groups)
RAPIDO (2020) [7, 96]	Randomised, international, multicentre Phase III	912	High-risk rectal adenocarcinoma (one of): • cT4a or cT4b • Extramural vascular invasion • Node stage: cN2 • Involved mesorectal fascia • Enlarged lateral lymph nodes	**TNT:** SCRT (5x5Gy) + 6 cycles CAPOX or 9 cycles FOLFOX then TME	Capecitabine-based CRT (50Gy or 50.4Gy) then TME Adjuvant: 8 cycles CAPOX or 12 cycles FOLFOX	Serious adverse events: 38% vs 34% Toxicity ≥ grade 3: 48% vs 35% Compliance: 84% vs 58% No difference in resection rates or post-op complications. R0 90% in both groups.	pCR: 28.4% vs 14.3% (*P*<0.0001) 3Y DRTF: 23.7% vs 30.4% (*P* = 0.019) 3Y OS: 89.1% vs 88.8% (NS)
PRODIGE 23 (2022) [8, 97]	Randomised, Phase III	461	cT3 or cT4 rectal adenocarcinoma	**TNT:** 6 cycles mFOLFIRINOX + CRT (50 Gy + capecitabine) then TME Adjuvant chemotherapy: 6 cycles FOLFOX6 or 4 cycles capecitabine	CRT (50.4Gy + capecitabine) then TME Adjuvant chemotherapy: 12 cycles mFOXFOX6 or 8 cycles capecitabine	Serious adverse events: 27% vs 22% (NS) Progression to surgery: 92% vs 95% (NS) (more palliative surgery in CRT group) R0: 95% vs 94% (NS) Post-op complications: 29.3% vs 31.2% (NS)	pCR: 28% vs 12% (*P*<0.0001) 3Y DFS: 75.7% vs 68.5% (*P* = 0.034) 3Y OS: 90.8% vs 87.7% (NS) Median OS (months): 76.3 vs 71.9 (*P* = 0.033)
CAO/ARO/AIO-12 (2022) [98, 99]	Randomised, Phase II	306	cT3 or T4 or cN+ rectal adenocarcinoma	3 cycles FOLFOX then fluorouracil/oxaliplatin CRT (50.4Gy) followed by TME	Fluorouracil/oxaliplatin CRT (50.4Gy) then 3 cycles FOLFOX followed by TME	Grade 3 or 4 toxicity: 37% vs 27% Compliance with CRT: 91% vs 97% Compliance with chemo: 92% vs 85% Surgical complications: 46% vs 35%	pCR: 19% vs 27% 3Y DFS: 73% both groups Interval from end of CRT to surgery (days): 45 vs 90 → no increase in surgical morbidity
OPRA (2022) [20]	Randomised, Phase II	324	Stage II & III rectal cancer (T3/4, N+)	Induction FOLFOX/CAPOX (16-18 weeks) + CRT (50-56Gy + fluorouracil or capecitabine) then TME or watch-and-wait	CRT (50-56Gy + fluorouracil or capecitabine) + consolidation FOLFOX/CAPOX (16-18 weeks) then TME or watch-and-wait	3Y TME-free survival: 41% vs 53%	pCR: 17% (induction) vs 25% (consolidation) 3Y DFS: 76% in both groups

*TNT* total neoadjuvant therapy, *SCRT short-course* radiotherapy, *CRT* chemoradiotherapy, *TME* total mesorectal excision.

Current evidence for TNT in oesophageal adenocarcinoma is limited to retrospective or small pilot study evidence but is promising and summarised in Table 2. Due to the non-randomised and largely retrospective nature of these studies, it is difficult to draw strong conclusions, however, there appears to be a consistently high disease-free and overall survival with intensified neoadjuvant regimens compared to standard of care. In addition, there are high rates of treatment completion and surgical resection as evidenced by in patients receiving FLOXFOX + CROSS [18]. Although there was an 80% grade 4 toxicity rate in the prospective study by Wo et al., the majority of this was due to subclinical lymphopenia. When patients with M1 nodal disease were excluded, 2-year progression-free survival was 78% in this study [19]. There are several ongoing trials of TNT or enhanced neoadjuvant therapy in patients with oesophageal or oesophagogastric junction adenocarcinoma. These are summarised in Table 3.

**Table 2:** Current evidence for total neoadjuvant therapy oesophagogastric adenocarcinoma.

Author	Study type	Patients (*n*)	Treatment	Survival outcomes	Other outcomes
NeoFLOT (2015) [100, 101]	Prospective, single arm	50	FLOT (x6)	Median DFS: 32.9 months 1 y OS: 79.3%	Febrile neutropenia: 1.7% ≥ grade 3 neutropenia: 29.3% ≥ grade 3 diarrhoea: 12.1% R0 rate: 86% pCR: 20%, TRG 1a/b: 40% Dose reduction: 43.1% of patients
Ho et al. [102]	Retrospective, comparative	580	Induction chemo + nCRT vs nCRT	Median OS: 3.38y vs 2.45y (*P* = 0.001)	ypT0N0: 21.2% vs 16.9% (NS)
Jurkowski et al. [103]	Retrospective, single arm	59	Induction chemo + nCRT	Median DFS: 3.5y Median OS: 5.8y 3-y OS: 72%	R0 rate: 89% pCR: 18.9%
Wo et al. [19]	Prospective, single arm	25	nFOLFIRINOX (x8) + nCRT	2y PFS: 55% 2y OS: 72%	TNT completion: 88% Surgical resection: 80% rate: 100% pCR: 35%
Dunne et al. [18]	Prospective, single arm	41	FOLFOX (x3) + CROSS	2y RFS: 71.5% Median RFS: 3.1y Median OS: not reached	95% completed nCT 98% completed CRT 87.7% underwent resection R0 rate: 97% pCR 22%
Carr et al. [104]	Retrospective, comparative	451	Induction FOLFOX + nCRT vs nCRT	2y DFS: 68% vs 44% (*P*<0.001)	pCR: 33% vs 22% (NS) Post-op complications: no difference

**Table 3:** Ongoing trials of total neoadjuvant therapy in oesophageal or gastroesophageal junction adenocarcinoma.

Reference	Study type	Patients (*n*)	Inclusion	Treatment					Reported outcomes	Awaited outcomes
TOPGEAR [105]	Randomised, international, multicentre Phase III	574	Gastric or junctional (Siewert II or III) adenocarcinoma T3/4 or N+, operable	2 cycles ECF + 5-FU & capecitabine-based CRT (45Gy) → surgery		3 cycles ECF → surgery			Compliance (preoperative): 98% vs 93% Compliance (postoperative): 53% vs 65% Progression to surgery: 85% vs 90% Surgical complications ≥ grade 3: 22% (both groups) GI toxicity ≥ grade 3: 30% vs 32% Haematologic toxicity ≥ grade 3: 52% vs 50%	Primary: Overall survival Secondary: DFS, pCR, toxicity, R0 rate
				Adjuvant: 3 cycles ECF						
CRITICS-II [106]	Randomised, Phase II, multicentre	207	Stage IB – IIIC, resectable gastric/ GOJ adenocarcinoma	Preoperative chemotherapy: 4 cycles DOC → surgery	Preoperative chemotherapy + CRT: 2 cycles DOC + CROSS → surgery			Preoperative CRT: CROSS → surgery	N/A	Primary: event-free survival Secondary: Time to event, time to recurrence, toxicity, surgical outcomes, RO rate, pCR, OS, QoL
TNT-OES-1 [107]	Phase II, single centre, single arm	20	Resectable primary oesophageal/GOJ (Siewert I or II) adenocarcinoma with oligometastatic disease^a^	4 cycles FLOT + CROSS CRT → surgery					N/A	Primary: tolerability Secondary: disease control rate, objective response rate, OS, PFS, toxicity, QoL, surgical outcomes
Shi et al. [108]	Phase II, multicentre, single arm	82	cT3/4, N+ locally advanced gastric/Siewert II/III adenocarcinoma	CRT (45Gy with oral S-1) + 6 cycles SOX → surgery					N/A	Primary: pCR Secondary: Toxicity, surgical complications, tumour downstaging, R0 rate,
RACE [109]	Randomised, multicentre, Phase III	340	Siewert I–III adenocarcinoma, cT3/4, any N or cT2N+ (M0)	4 cycles FLOT		2 cycles FLOT + CRT (45Gy + 5-FU + oxaliplatin)			N/A	Primary: PFS Secondary: OS, R0 rate, number of harvested LNs, site of tumour relapse, QoL
				4 cycles adjuvant FLOT						
PREACT [110]	Randomised, multicentre, Phase III	682	Gastric or Siewert II/III adenocarcinoma. Stage IIB–IIIC	1 cycle S-1 + oxaliplatin → CRT (45Gy + S-1) → 1 cycle S-1 → surgery			3 cycles S-1 + oxaliplatin → surgery		N/A	Primary: DFS Secondary: OS, R0 rate, pCR, toxicity, surgical complications
				3 cycles adjuvant S-1 + oxaliplatin						
https://classic.clinicaltrials.gov/ct2/show/NCT04028167	Phase II, multicentre, single arm	40	cT3/4 or N+ oesophageal or GOJ adenocarcinoma	FLOT + CRT					N/A	Primary: pCR Secondary: 1Y OS & DFS, toxicity, QoL, change in SUVma (CT PET), ctDNA

*DOC* docetaxel + oxaliplatin + capecitabine, *SOX* S-1 + oxaliplatin.

^a^Maximum of four resectable metastatic lesions or lesions suitable for stereotactic irradiation. In a maximum of two organs.

The, albeit limited, existing evidence in oesophageal and junctional adenocarcinoma, as well as other solid tumours suggests a significant survival benefit for patients receiving TNT and allows more patients to complete all oncological therapy and surgery. Prospective, randomised controlled trials are needed to compare treatment modalities directly using the TNT approach in oesophageal cancer.

## Total neoadjuvant therapy—assessing treatment response

If patients are to embark on a prolonged course of preoperative treatment, ideally it should be a precision medicine strategy with mechanisms in place for an as early as possible assessment of response and adaption of treatment accordingly. Again, rectal cancer might help us to address this problem. The OPRA trial compared surgical resection to a “watch-and-wait” approach following TNT in patients with locally advanced rectal cancer [20]. This trial used an extensive surveillance protocol with a combination of digital rectal examination, flexible sigmoidoscopy (every 4 months for the first 2 years, then 6 monthly), CEA, MRI, CT chest/abdomen/pelvis and colonoscopies at year 1 and year 5 and demonstrated that organ preservation is safe and achievable in half of patients. Findings from the International Watch & Wait Database emphasise the importance of endoscopic surveillance [21]. We can also look to oesophageal squamous cell carcinoma, for which definitive chemoradiotherapy (dCRT) is a treatment option. Surveillance after dCRT includes regular OGD and biopsies, EUS, CT scan and Fluorine 18 fluorodeoxyglucose (FDG) positron emission tomography (PET-CT) [22, 23]. The SANO trial is ongoing, which investigates the use of active surveillance in an organ-sparing “watch-and wait” approach for patients with oesophageal squamous cell and adenocarcinoma and utilises PET-CT, OGD with biopsies and EUS with FNA of suspicious nodes in its clinical response evaluation [24].

Generally, PET-CT has been shown to be a feasible and accurate modality for detecting response to neoadjuvant therapy in oesophageal cancer [25–28]. However, its accuracy in detecting non-response is questionable, with one study suggesting reliable detection of non-responders in gastric and Siewert II-III cancers [29], and another suggesting a lack of accuracy in detecting non-response in oesophageal cancer [30]. In the absence of a “one size fits all” endoscopic or radiological surveillance modality and a lack of reliable tumour markers in oesophageal adenocarcinoma, it may be that a multimodality approach is required to assess response, and more importantly, non-response to preoperative treatment. The burden of such an approach for patients and the health care system will need to be understood.

Circulating tumour DNA (ctDNA) is well established as a marker of minimal residual disease and correlates with recurrence and survival in patients undergoing neoadjuvant therapy for rectal cancer [31–36]. The use of ctDNA in oesophageal cancer is still being established. The prospective pilot study of TNT in oesophageal adenocarcinoma analysed ctDNA at various time points, including post-chemoradiotherapy and post-surgery [19]. Those with undetectable ctDNA post-chemoradiotherapy and post-operatively had significantly lower recurrence rates compared to those with detectable ctDNA at these time points (8% vs 75% post-CRT, p = 0.004; 0% vs 40% post-op, *P* = 0.045). ctDNA has been identified in other studies as a useful biomarker of recurrence and treatment response in oesophageal cancer [37–39]. This highlights the potential utility of ctDNA as a biomarker of response to treatment, a predictor of recurrence and its utility in planning adjuvant treatment where needed. It is essential that any future trials of TNT incorporate several modalities to monitor response to treatment such as PET-CT, endoscopic surveillance and ctDNA.

## Surgical resection margin

The importance of complete surgical excision of oesophageal and gastroesophageal junction (GOJ) cancers is long established. The 3-year survival in those with a complete surgical resection (R0) in OEO2 was 42.4% compared to 18.0% and 8.6% in those with R1 and R2 resections, respectively [40]. Patients who undergo preoperative oncological therapy are more likely to have an R0 resection. This is particularly evident in regimens where all the therapy is delivered pre-operatively, for example, in the CROSS trial, those receiving preoperative chemoradiotherapy had an R0 rate of 92% compared with 69% in those having surgery alone [5]. Neoadjuvant chemoradiotherapy has been shown to deliver better local tumour control (R0 resection rate) than preoperative chemotherapy. In the Neo-AEGIS study, which compared neoadjuvant CRT (CROSS) with perioperative chemotherapy, R0 in the CROSS group was 95% compared to 82% in the perioperative chemotherapy group [41]. Similar results have been shown for R0 rate in the NeoRes I study, where chemoradiotherapy (R0=89%) and chemotherapy (R0=71%) were compared in the neoadjuvant setting alone in patients with T1-T3 disease[42]. However, there were comparable R0 rates between nCRT and nCT in the POET study (72% vs 69%), in patients with T3-T4 disease [43]. It should be noted that in these comparative studies older chemotherapy regimens were largely used in the chemotherapy arm rather than FLOT. The ESOPEC trial is currently ongoing, which directly compares FLOT and CROSS [44]. In the FLOT4 trial, where 83% of patients who received perioperative FLOT chemotherapy had T3/T4 disease and 78% had node-positive disease, there was an R0 resection rate of 92% in those with resected specimens [45]. The recent DANTE trial, in which 93% of patients completed pre-op FLOT cycles, had similarly high R0 rates of 91% in the FLOT arm and 92% in FLOT + atezolizumab [46]. Indirect comparison of the studies above is limited by significant differences in the study population, notably differences in histological type, disease location, disease stage, age, and performance status. This highlights the need for precise patient selection in clinical trials comparing treatment modalities.

From the available evidence, if the objective of preoperative treatment were solely to improve R0 resection rate both CROSS and FLOT offer comparable outcomes, but other important outcomes that have a profound impact on overall survival need to be considered.

## Lymph node status, primary tumour pathological response and survival

Systemic disease control in patients with node-positive or micrometastatic disease is important for improving long-term outcomes in patients with oesophageal cancer [47–55]. A recent meta-analysis has highlighted the importance of lymph node downstaging after neoadjuvant therapy as a prognostic factor in oesophageal cancer, with those with ypN0 achieving a much-improved survival over those with positive nodes (ypN+) after neoadjuvant therapy [56]. The POET study demonstrated a significantly improved 3-year survival for patients with an R0 resection and ypN0 (64.2%) compared with those who had tumour in the resected lymph nodes (38.8%), *P* < 0.001 [43]. Other studies have demonstrated the benefit of ypN0 as a prognostic factor in surgery for oesophageal cancer [57], with one study showing response in the lymph nodes and primary tumour to independently improve disease-free survival [58]. Two studies have suggested that adequate lymph node response improves survival, even if there is little response in the primary tumour [59, 60]. Furthermore, lymph node status was the largest determinant of prognosis in a recent machine-learning model predicting long-term survival [61]. Although there is a survival benefit for ypN0 over those with positive lymph nodes in the resected specimen (ypN+), the greatest benefit is seen in those with natural N0 or in those in whom there is concomitant complete regression in the primary tumour (ypT0)[62]. This highlights the importance of adequate pathological response in both the primary tumour and the lymph nodes. Moreover, primary tumour pathological complete response (pCR) has demonstrated 5-year overall survival of 88% vs 39% in those with complete resection (R0) but residual tumour in the resected specimen [63]. In a separate study, pCR was demonstrated as an independent predictor of improved survival following neoadjuvant chemoradiotherapy [64]. However, the Neo-AEGIS study, which compared neoadjuvant chemoradiotherapy to mostly older ECX perioperative chemotherapy, demonstrated higher rates of pCR (16% vs 5%) and ypN0 (60.1% vs 44.5%) after nCRT compared to nCT but this did not translate into improved survival [41]. If the objective of preoperative treatment were solely to improve pCR in the primary tumour and lymph nodes, neoadjuvant chemoradiotherapy would be the clear treatment of choice. However, it is important to consider how this impacts disease-free and overall survival.

## Adjuvant therapy

The evidence for adjuvant therapy alone is extremely limited in gastroesophageal adenocarcinoma and is restricted to trials in gastric and gastroesophageal junction adenocarcinoma [65, 66]. In oesophageal adenocarcinoma, specific benefit of adjuvant therapy has only been demonstrated within the context of perioperative chemotherapy. In a large retrospective analysis, patients who received adjuvant chemotherapy had improved median survival over those who did not receive adjuvant chemotherapy (62.7 months vs 50.4 months) [67]. Moreover, the benefit of completing all cycles of FLOT has been shown to improve overall survival, regardless of tumour regression [68]. Adjuvant chemotherapy is also associated with improved median overall survival (40 months vs 34 months) in patients who had preoperative chemoradiotherapy [69]. Other studies suggest that the benefit of adjuvant chemotherapy in this setting is greatest in node-positive disease [70, 71]. Indeed, in their subgroup analysis in patients receiving perioperative chemotherapy Rahman et al. [67] found that patients who had ypN0 had excellent survival outcomes, with no additional benefit from adjuvant chemotherapy whereas those with ypN+ had superior survival if they received adjuvant chemotherapy. The relative benefit of neoadjuvant over adjuvant therapy alone has been shown in patients with gastric cancer in the PRODIGY trial with increased 3-year PFS in those receiving nCT (66.3%) compared to adjuvant CT alone (60.2%) [72]. Retrospective studies also show survival benefit in those with gastro-oesophageal junction adenocarcinoma receiving nCRT over adjuvant CRT [73].

Adjuvant therapy following preoperative oncological therapy can improve survival outcomes for patients with oesophageal adenocarcinoma, particularly those with residual nodal disease and is part of the current standard of care. However, there are challenges in delivering adjuvant chemotherapy in patients who have undergone oesophagogastrectomy and there is some evidence to suggest a relative benefit of neoadjuvant therapy over adjuvant.

## Current standard of care and its challenges

Although accepted as a standard of care, the perioperative approach of neoadjuvant chemotherapy followed by surgery and adjuvant chemotherapy thereafter is often hampered by failure to complete all chemotherapy cycles. In the FLOT4 trial, only 46% of patients completed all cycles using the perioperative approach [4]. Whereas, in regimens where all treatment is delivered pre-operatively there is a much higher rate of treatment completion without reducing the number of patients proceeding to surgery. An example is the CROSS trial, in which 95% of patients completed oncological treatment and 90% of patients underwent resection, albeit that the amount of chemotherapy delivered in this regimen is much less than in FLOT. However, in the FLOT4 trial, 90% of patients completed all preoperative chemotherapy, suggesting that preoperative treatment is better tolerated than postoperative treatment, in part due to the morbidity following oesophagogastrectomy. Timing of surgery after neoadjuvant therapy is also an important consideration. Patients undergoing nCRT have improved response after delayed surgery (>7–8 weeks after nCRT completion) but have higher 30-day mortality after surgery [74]. A large study of >2000 patients suggests that the optimal timing for surgery is 56 days after nCRT completion to balance increased pathological response with overall survival [75].

Both the perioperative FLOT chemotherapy regimen and the preoperative CROSS chemoradiotherapy regimen plus surgery are accepted standards of care for patients with resectable oesophageal adenocarcinoma and are currently being compared in the ESOPEC trial [44]. Although there is currently no directly comparable clinical evidence to suggest that either is superior to the other, CROSS (nCRT) and FLOT (perioperative chemotherapy) have different effects on the primary tumour and systemic disease. There is a higher rate of pCR with CROSS than preoperative chemotherapy. Due to its radiotherapy component, CROSS gives the opportunity to downstage primary tumours where there is a risk of R1 resection. However, CROSS delivers less systemic treatment than FLOT. As a result, there is a risk of systemic undertreatment in patients allocated to nCRT using CROSS. This has been demonstrated in a recent large cohort study of patients with oesophageal adenocarcinoma all achieving pCR (ypT0N0) after neoadjuvant therapy, which showed that 5-year recurrence-free survival was significantly better in the nCT group (87.1%) compared to nCRT (75.3%), notably with a greater prevalence of distant recurrence in the nCRT group, suggesting potential systemic undertreatment [55]. This has also been demonstrated in 10-year CROSS follow-up, in which CROSS reduced oesophageal cancer-related death by reducing locoregional recurrence but did not reduce the incidence of distant recurrence compared to surgery alone [76].

Regarding pCR in the primary tumour and lymph nodes (ypT0N0), it is important to consider how this translates to into long-term outcomes and the differential outcomes observed after different neoadjuvant regimens. It is evident that whilst achieving ypT0N0 is important, the modality used to achieve this is also important for survival outcomes. Although there were higher rates of ypT0 and ypN0 in those receiving nCRT compared to nCT (using older ECX chemotherapy rather than FLOT) in the NEO-AEGIS study, this did not translate into improved 3-year overall survival (56% vs 57%) [41]. There were similar results in NeoRes I, with higher pCR for nCRT than nCT (28% vs 9%) but similar 5-year OS (42.2% vs 39.6%) [42]. A recent retrospective study directly comparing FLOT vs CROSS shows similar 5-year overall survival in patients receiving FLOT and CROSS despite a higher pCR with CROSS [77]. Other recent smaller studies demonstrate similar survival patterns between CROSS and FLOT but show higher distant recurrence and postoperative respiratory failure with CROSS [78, 79]. Results from the ESOPEC Phase III trial are eagerly awaited [44]. A 2019 meta-analysis makes the conclusion that although the addition of radiotherapy to chemotherapy alone increases the chance of pCR and reduces the risk of locoregional failure, it does not reduce the risk of distant metastases or death [80].

These clinical observations support tumour biology relating to intra-patient heterogeneity. One study has shown discrepancy in genomic alterations between primary tumour and metastatic disease and highlights the limitations of using genetic alterations in biopsies of the primary tumour to guide treatment in other areas of the patient’s disease such as distant metastases [81]. Furthermore, intratumoural heterogeneity exists between tissue from superficial primary tumour, deep primary tumour, and lymph node metastases [82].

The main challenges for the current standards of care are non-completion of perioperative therapy in the context of FLOT, as well as a risk of systemic undertreatment in those receiving CROSS. Whilst pCR is seen as a marker of treatment success, studies comparing patients achieving pCR who received nCT or nCRT lead us to conclude that ypT0N0 does not always translate into the same outcomes in primary endpoints such as disease-free or overall survival between treatment modalities and should not be used as a surrogate primary endpoint. Future comparative randomised trials should focus not only on pCR but also on survival outcomes. By combining both modalities in the preoperative setting using a total neoadjuvant approach, for example using extended preoperative FLOT or a combination of preoperative FLOT plus CROSS, we may be able to achieve both optimal locoregional and systemic disease control, without compromising progression to surgery, enabling more patients to complete all intended treatment.

## The role of immune checkpoint inhibitors

Immune checkpoint inhibitors remove the inhibitory signals of T-cell activation that enable tumour-reactive T cells to overcome regulatory mechanisms and mount an effective antitumour response [83]. Although their mechanism of action is different, there are synergies between chemotherapy and immunotherapy and it has been suggested that an effective strategy to harness such synergies is to give immune checkpoint inhibitors after the tumour mass has been optimally reduced with surgery and systemic chemotherapy in the setting of minimal residual disease, where the negative impact of tumour bulk on antitumour immune response is minimised [84]. In the context of TNT, this could theoretically enable best possible response to local and systemic therapy whilst engaging the immune response in the postoperative setting. The positive impact of postoperative checkpoint inhibitors has been demonstrated in the nCRT setting in the CHECKMATE 577 trial, in which patients with residual disease after surgery (ypT+ or ypN+) were randomised to receive adjuvant PD-1 inhibitor, nivolumab, or placebo [85]. Median disease-free survival was 22.4 months in the treatment vs 11 months in the placebo arm (HR 0.69, *P* < 0.001). These results have changed the paradigm of treatment for oesophageal cancer, giving us a fourth treatment modality in addition to chemotherapy, radiotherapy, and surgery to improve outcomes for patients with locally advanced, high-risk oesophageal cancer. It must be noted that quality of life scores were comparable between the placebo and treatment groups with an acceptable safety profile, which is important when considering patients who might have already received TNT and surgical resection for further treatment [86]. However, recent trial results have failed to show a benefit for the addition of immune checkpoint inhibitors to neoadjuvant and adjuvant chemotherapy. Final results are awaited but KEYNOTE-585 reports a higher pathological complete response rate from the addition of pembrolizumab to perioperative FLOT, but the event-free and overall survival endpoints were not met [87]. Similarly, ATTRACTION-5 reported no recurrence-free survival benefit from the addition of Nivolumab to adjuvant chemotherapy [88]. In both KEYNOTE-585 and ATTRACTION-5 the use of immune checkpoint inhibitors was in biomarker unselected patients, and it is relevant that in CHECKMATE577 a post hoc subgroup analysis indicated that disease-free survival benefit from adjuvant nivolumab was only demonstrated in PDL1 combined positive score (CPS) ≥5 patients and not seen in PDL1 CPS ≤5. In metastatic or advanced-stage unresectable gastroesophageal cancer patients a number of randomised trials have reported the benefit of the addition of immune checkpoint inhibitor to chemotherapy and tumour PDL1 CPS has demonstrated benefit as a biomarker to predict the quantum of benefit from the checkpoint inhibitor. In CHECKMATE 649, patients with metastatic, or unresectable oesophageal, junctional or gastric adenocarcinomas who were not known to be HER2 positive were enrolled regardless of PDL1 CPS result, but the co-primary endpoints were PFS and OS in patients with PDL1 CPS ≥5 where benefit was seen with the addition of nivolumab to oxaliplatin plus capecitabine or 5-FU (OS HR = 0.70 (95% CI 0.61, 0.81), PFS HR = 0.70 (95% CI 0.60, 0.81)) [89]. Similarly in KEYNOTE 859, in metastatic or advanced-stage unresectable gastroesophageal junctional or gastric adenocarcinomas OS and PFS benefit from the addition of pembrolizumab to platinum fluoropyrimidine chemotherapy was demonstrated recently in patients with PDL1 CPS ≥1 with a greater incremental benefit seen in those with PDL1 CPS ≥10 [90]. While KEYNOTE 590 demonstrated benefit of the addition of pembrolizumab to platinum fluoropyrimidine chemotherapy to all randomised patients with oesophageal cancer (squamous and adenocarcinoma) and Siewert type I junctional adenocarcinomas, but greater incremental benefit in those with PDL1 CPS ≥10 [91]. These trials demonstrating the survival benefit of checkpoint inhibitors in unresectable and metastatic gastro-oesophageal malignancy have established new standards of care in biomarker-selected patients with advanced-stage disease and underscore the importance of biomarker-directed use of immune checkpoint inhibitors. Together with the recent results from KEYNOTE-585 and ATTRACTION-5, and post hoc PDL1 CPS analysis from CHECKMATE 577 this suggests that biomarker section for immune checkpoint inhibitors is likely to be important in the neoadjuvant and adjuvant setting as well. This has important relevance for ongoing trials of immune checkpoint inhibitors in the curative setting, including those which incorporate perioperative FLOT such as MATERHORN (FLOT + durvalumab or placebo) (NCT04592913). Microsatellite instability-high (MSI-H) is present in 6–24% of resected gastroesophageal adenocarcinoma and is an established predictive biomarker for immune checkpoint inhibitors [92]. Encouraging results have been reported in non-randomised Phase 2 trials of perioperative immune checkpoint inhibitors without chemotherapy in MSI-H selected patients, for example, the NEONIPIGA trial has shown pCR rates of 59% in patients with MSI-high disease, but survival follow-up is limited at present and larger randomised studies are yet to be undertaken [93].

Overall, the role of immune checkpoint inhibitors in perioperative treatment of gastroesophageal adenocarcinomas is not yet established and emerging trial results highlight the importance of biomarker-directed use of these agents. This emphasises the importance of optimising the conventional perioperative treatments with chemotherapy and chemoradiotherapy for those patients who are immune checkpoint inhibitor biomarker negative and the incorporation of treatment with checkpoint inhibitors in a biomarker-directed manner into trials of TNT where patients have residual disease despite optimal local and systematic therapy.

## Conclusion

In Summary, the addition of perioperative oncological therapies to surgery have greatly improved overall and progression-free survival in patients with oesophageal and junctional adenocarcinoma, achieving higher R0 resection rates and pathological response in the primary tumour and involved lymph nodes. However, more than 50% of patients do not complete all planned therapy if receiving perioperative chemotherapy. Furthermore, whilst pCR is important, intra-tumour heterogeneity impacts how this translates into long-term disease-free survival, and the impact of FLOT and CROSS on survival does not appear to be directly related to pCR alone. Total neoadjuvant therapy has shown promising results with high pCR rates together with impressive disease-free and overall survival in the retrospective setting. This warrants a randomised controlled trial of total neoadjuvant therapy approaches, incorporating methods of treatment response.
